# FluoAnalysis: An Open-Source MATLAB Toolbox for Analysis of Calcium Imaging Measurements of Oscillatory Astrocytic and Neuronal Networks

**DOI:** 10.3390/brainsci14080830

**Published:** 2024-08-19

**Authors:** Márton Péter, László Héja

**Affiliations:** 1 Institute of Organic Chemistry, HUN-REN Research Centre for Natural Sciences, Magyar Tudósok Körútja 2, 1117 Budapest, Hungary; peter.marton@ttk.hu; 2 Hevesy György PhD School of Chemistry, ELTE Eötvös Loránd University, 1117 Budapest, Hungary

**Keywords:** astrocytes, neurons, neural activity, software, two-photon, electrophysiology, oscillations, spectral analysis

## Abstract

Calcium imaging, especially two-photon imaging, has become essential in neuroscience for studying neuronal and astrocytic activity under in vivo and in vitro conditions. Current advances in the development of calcium sensors as well as imaging hardware enable high-frequency measurements of calcium signals in hundreds of cells simultaneously. The analysis of these large datasets requires special tools and usually a certain level of programming experience. Despite advancements in calcium imaging analysis software development, significant gaps remain, particularly for data acquired at a high sampling rate that would allow for the spectral analysis of calcium signals. The FluoAnalysis MATLAB toolbox addresses these gaps by offering a comprehensive solution for analyzing simultaneously measured calcium imaging and electrophysiological data. It features both GUI-based and command-line approaches, emphasizing frequency domain analysis to reveal network-level oscillatory signals linked to single-cell activity. In addition, the toolbox puts special emphasis on differentiating between astrocytes and neurons, revealing the interactions between the network activity of the two major cell types of the brain. It facilitates a streamlined workflow for data loading, ROI identification, cell classification, fluorescence intensity calculation, spectral analysis, and report generation, supporting both manual and automated high-throughput analysis. This versatile platform enables the comprehensive analysis of large imaging datasets. In conclusion, the FluoAnalysis MATLAB toolbox provides a robust and versatile platform for the integrated analysis of calcium imaging and electrophysiological data, supporting diverse neuroscience research applications.

## 1. Introduction

Calcium imaging, especially two-photon imaging, has emerged as a pivotal technique in neuroscience for studying neuronal activity and dynamics under both in vivo and in vitro conditions. This method allows for the visualization and quantification of calcium signals in a large number of cells simultaneously, providing valuable insights into neural communication, signal transduction, and the overall functional architecture of neural circuits [1]. In addition to neurons, calcium imaging is particularly suitable to study the activity of astrocytes, the major non-excitable cells of the brain that play indispensable roles in a multitude of physiological and pathophysiological brain functions. Despite significant advancements and the recent release of decent calcium imaging analysis packages [2,3,4,5,6,7], there remain critical gaps in the software tools available for the analysis of calcium signals [8], particularly in the context of astrocytes that are often neglected due to the lack of specialized software for analyzing their activity.

Another critical limitation is the absence of software capable of performing a parallel analysis of imaging and electrophysiological data. Integrating these two types of data is crucial for a comprehensive understanding of cellular and network activity, as calcium imaging and electrophysiological data provide complementary insights into cellular function. Electrophysiological recordings offer a precise temporal resolution, while calcium imaging provides the spatial context. However, most available software solutions do not support a parallel analysis, forcing researchers to rely on separate, often incompatible tools for each data type [9].

Furthermore, the available analysis tools primarily focus on detecting single events (peaks) in calcium signals [10,11], paying much less attention to revealing the spectral components of the oscillating calcium signals that may provide deeper insights into the network dynamics of large-scale neuronal and astrocytic ensembles and decipher the underlying biological processes. Notably, the spectral analysis of electrophysiological signals has previously led to ground-breaking findings about neuronal oscillations and their roles in various vital physiological processes [12]. Despite the success of the spectral analysis of electrophysiological recordings, however, the current analysis tools lack robust functionality for decomposing and analyzing different frequency components in imaging data, which may be even more relevant when considering the high-frequency calcium fluctuations in thin astrocytic processes [13]. This limitation hampers our ability to fully interpret the complex dynamics of calcium signaling [14].

To address these gaps, we introduce a comprehensive analysis software for calcium imaging data. The FluoAnalysis MATLAB toolbox provides both GUI-based and command-line approaches for the simultaneous analysis of calcium imaging and electrophysiological data. In addition, the toolbox puts special focus on investigating the frequency domain of the imaging results for revealing network-level oscillatory signals that can be attributed to single-cell activity in order to fully utilize the advantages of the imaging methodology.

## 2. Results

The FluoAnalysis MATLAB toolbox is controlled by well-organized MATLAB classes. Most of the functions in these classes can be called from user-friendly GUIs as well. The analysis workflow consists of the following modules: (1) loading of the imaging data file, (2) loading of the corresponding electrophysiological recording, (3) identification of regions of interests (ROIs), (4) classification of identified ROIs as neurons or astrocytes, either automatically or manually, (5) calculation of the ΔF/F_0_ traces from the imaging data, (6) spectral analysis of the imaging and electrophysiological signals, and (7) creating detailed reports in a Word format. The workflow developed with the GUI can used for automated analysis in the non-interacting mode.

### 2.1. Loading Calcium Imaging Data

The ‘loadImage’ function is the primary component of the FluoAnalysis toolbox. This function is designed to import various image file formats, including multipage .TIFF files, .MES and .MESC files of the MES software package for Femtonics two-photon microscopes, as well as previously analyzed .MAT files in MATLAB format. For .TIFF files, the function gathers metadata to ascertain image dimensions, frame count, and channel number, subsequently loading the data into a multidimensional array. The .MES and .MESC files are processed with particular attention to their unique metadata structures. Additionally, the function can handle previously analyzed .MAT files, facilitating the integration of historical datasets into current analyses. Either frame- or line-scan images can be loaded into the software. Moreover, the ‘loadImage’ function can also load pre-defined ROI files created by the toolbox during automatic image acquisition on Femtonics two-photon microscopes.

**Figure 1:**
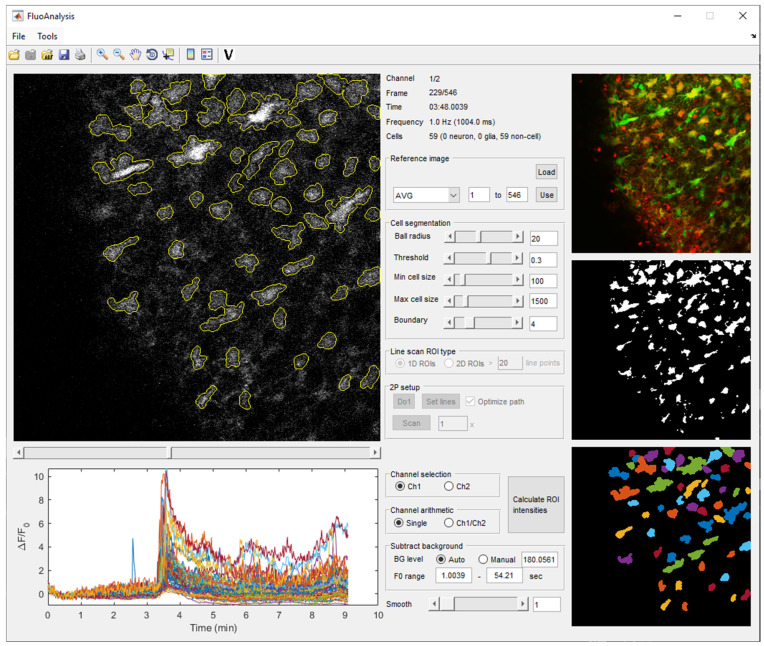
Main GUI of the FluoAnalysis toolbox. A multi-tiff file, imported into the GUI with the identified ROIs marked by yellow contours (**top left**). Average projection of the 546 imported frame is visible at the (**top right**), followed by a thresholded binary image and the ROIs in pseudo colors below. ROI detection and ΔF/F_0_ calculation parameters are located in the middle column. The calculated ΔF/F_0_ traces for all identified ROIs during a control period and application of 1 mM ATP to a hippocampal brain slice expressing GCaMP2 calcium-sensitive fluorescent protein in neurons and astrocytes are visible in the bottom left.

After the image file is loaded, it can be visualized and browsed by a slider to easily assess the dynamics of the fluorescent signal (Figure 1). Two independent channels can be displayed to explore signals from different fluorescent indicator dyes or proteins.

### 2.2. Loading Corresponding Electrophysiological Recording

The ‘loadEphys’ function is an integral part of our analysis software for synchronizing electrophysiological recordings with calcium imaging data. This function loads electrophysiological data from .ABF files and aligns it with the imaging session using the metadata in the .ABF file. If the electrophysiological recording was continuous during multiple imaging sessions, the function can also identify the electrophysiological data segment corresponding to a specific imaging session, leveraging tagged markers within the .ABF file when available. The extracted segment is downsampled by a factor of 10 and stored within the object, ensuring precise temporal alignment between electrophysiological and imaging data. This synchronization is crucial for the comprehensive analysis of neuronal activity, providing a robust framework for correlating electrophysiological and calcium signals.

### 2.3. Identifying Cells

The ‘findROIs’ function in the FluoAnalysis toolbox is designed to identify regions of interest (ROIs) on a reference image. The reference image can be either made by external tools and loaded by a GUI command or created from a given set of the series of frames applying the average, maximum, or standard deviation function to the selected frames. After loading or creating the reference image, various parameters can be set to threshold it and create a binary image from which objects are extracted and filtered based on specified cell size criteria. For line-scan data, the function maps the ROIs to line-scan positions and filters out objects with insufficient pixel representation. The identified ROIs are visualized in pseudo color on the GUI. This comprehensive approach ensures the accurate supervised detection and mapping of ROIs, crucial for analyzing cellular activity.

To facilitate the investigation of the same cells over multiple imaging sessions, the ROIs can be imported from a previously analyzed .MAT file using the ‘File > Load objects from another file’ GUI menu. To adjust for specimen or objective movements, the imported ROIs can be moved in the x–y plane using the ‘Tools > Move ROIs’ menu option.

### 2.4. Classification of Identified Cells as Neurons or Astrocytes

The FluoAnalysis toolbox is tailored to analyze calcium imaging data measured simultaneously in neurons and astrocytes. Therefore, it is crucial to appropriately classify cell types. The ‘autoClassifyCells’ function provides an automated classification method based on the ratio of green channel (calcium-sensitive dye or protein, e.g., Oregon Green BAPTA or GCaMP-6) and red channel (fluorescent astrocyte marker, e.g., SR-101) fluorescence. ROIs are first validated as cells if the ROI area is higher than a predefined threshold (100 pixels), the eccentricity of the ROI is lower than 0.85 (assuming that mostly the cell bodies are labelled), and the mean fluorescent intensity within the ROI is at least two times higher than the mean fluorescent intensity in the close vicinity of the ROI. The identified cells are further classified as astrocytes if they have sufficient area, appropriate eccentricity, and specific intensity ratios indicating glial characteristics. Conversely, neurons are identified based on distinct intensity profiles and ratios indicative of neuronal properties. This automated approach streamlines the identification and categorization of cellular elements in imaging datasets, enhancing the efficiency and accuracy of data analysis.

**Figure 2:**
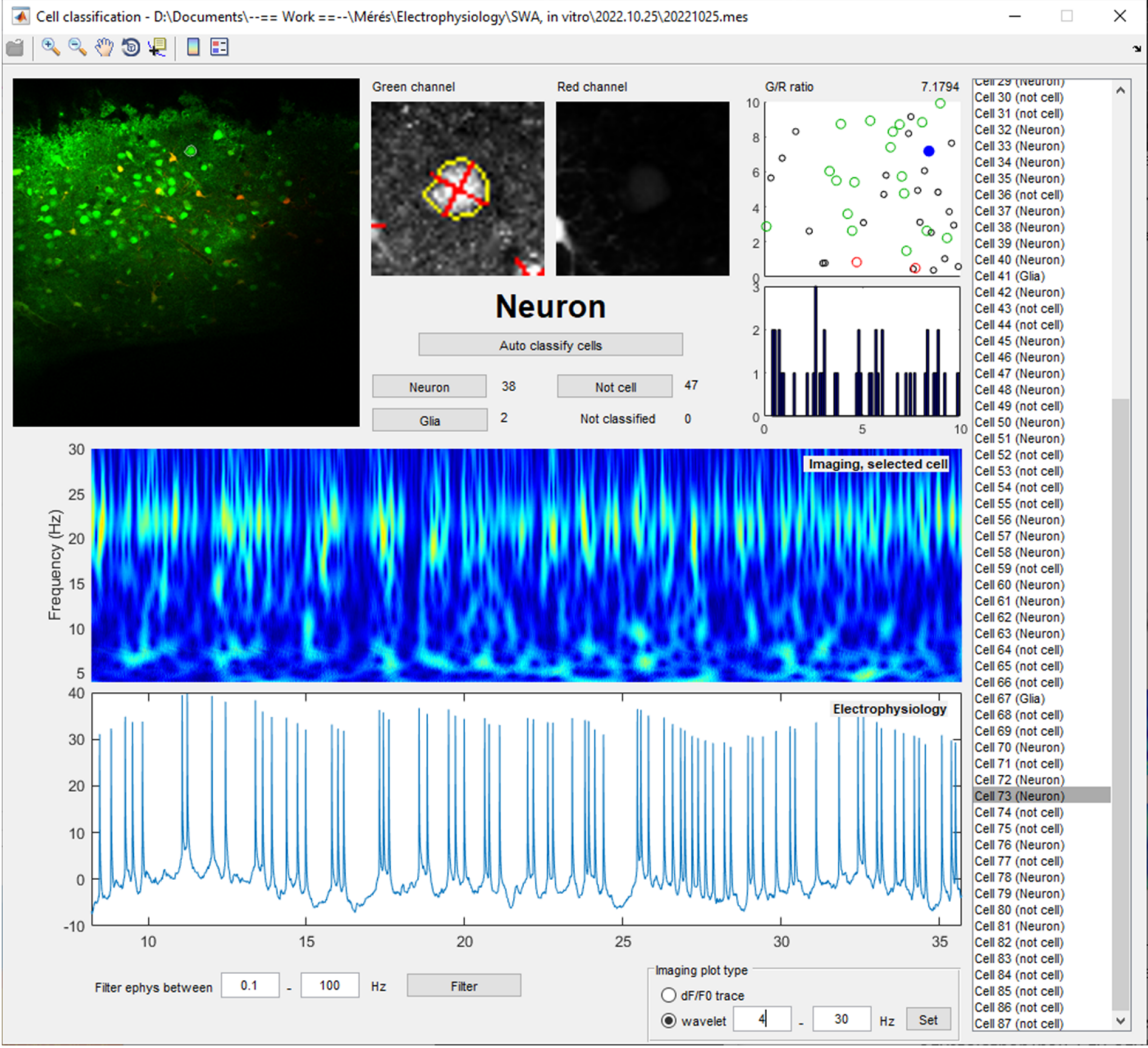
A GUI for classification and spectral analysis of Ca^2+^ imaging data of individual cells. High Ca^2+^ imaging sampling rate (68 Hz) together with the unique spectral analysis capabilities of the FluoAnalysis toolbox enables the identification of a high-frequency imaging signal that corresponds to the firing rate of a cortical pyramidal cell as measured by patch clamp electrophysiology. All imaged cells (listed on the right) can be individually investigated. Automatic classification of the cells as neurons or astrocytes, based on the ratio of green and red channel intensities in the ROIs, can be manually overridden. Wavelet analysis can be easily applied at different frequency bands.

The automatic classification can be replaced or overridden by manual cell identification using a GUI (Figure 2). The ‘Tools > Cell validation’ menu provides a user interface that displays the reference image, a high magnification view of the green and red channels of the ROIs, as well as the calculated ΔF/F_0_ traces for each cell to allow the experimenter to make an informative decision on the cell type.

Moreover, cells can be extracted as individual images by the ‘exportCells2DB’ function to be classified by external programs or used as a training set for machine learning approaches. The classification results can be imported back to the original dataset by the ‘importCellAnnotation’ function.

### 2.5. Analysis of Calcium Activity

Following ROI identification, cell validation, and cell type classification, the ‘calculateF’ and ‘calculateDeltaFperF0’ functions compute fluorescence intensity (F) and relative fluorescent intensity changes (ΔF/F_0_) within ROIs across multiple frames and channels from raw imaging data. Additionally, if a red channel is present, the green-to-red fluorescence ratio change (ΔG/R) is calculated as the fluorescent intensity change from the first frame in the green channel, divided by the fluorescent intensity of the red channel to provide a measure that can minimize potential movement artefacts. The user can choose between automatic or manual background correction and can also override the control range across which the F_0_ value is calculated. The GUI also provides a convenient way to smooth the resulting ΔF/F_0_ traces (Figure 1).

### 2.6. Wavelet Analysis

The FluoAnalysis toolbox puts special focus on the analysis of calcium imaging data acquired at a high sampling rate, which enables the identification of periodic activity in the physiologically relevant frequency ranges identified for neurons, like the delta (0.5–4 Hz), theta (4–8 Hz), alpha (8–13 Hz), or beta (13–30 Hz) frequency bands [12,15] that are correlated with distinct physiological and pathological functions. The ‘calculateWavelet’ function is designed to perform a wavelet analysis on both calcium imaging and electrophysiological data, providing insights into the dynamics of different frequency components of these signals. The function utilizes the continuous wavelet transform (CWT) with a complex Morlet wavelet to analyze the time series data. The normalized wavelet coefficients are stored as a function of both time and frequency, facilitating the subsequent analysis of temporal dynamics and frequency content. This dual approach allows for the simultaneous analysis of both calcium imaging and electrophysiological data within a unified framework and enables researchers to investigate and compare the temporal and frequency domain characteristics of neuronal and astrocytic activity (Figure 2).

### 2.7. Analysis of Network Synchronization

Since the FluoAnalysis toolbox is specifically tailored for the analysis of calcium activity simultaneously measured in large numbers of astrocytes or neurons, it provides different parameters that can be used to characterize network synchronization.

The ’calculatePhaselock’ function is designed to assess the phase synchrony between all cell pairs in the field of view. It computes the phase-locking value (PLV), a measure of the consistency in phase relationships between pairs of cells, which is crucial for understanding neural network dynamics. A specific frequency range can be set for the function in order to calculate phase synchronization in different frequency bands. The function also manages data edge effects by zeroing out the first and last segments of the phase data, thereby ensuring the accuracy of the PLV calculations. The results are stored in a symmetric matrix format, allowing for the straightforward interpretation and further analysis of cell-pair synchrony.

The ‘calculateCrossCorrelation’ function provides another measure of network synchrony. It computes the cross-correlation between ΔF/F_0_ traces of all cell pairs. Alternatively, it can calculate the auto-correlation of the ΔF/F_0_ trace of a single cell. It automatically handles differences in signal length and adjusts parameters such as step size and window size based on the sampling interval, ensuring consistency across different datasets. The function also offers flexibility in defining the time shift range within which the signal correlation is determined. This function calculates cross-correlation across sliding windows of the data, providing a time-resolved analysis of signal synchronization. The results include the maximum correlation values and the corresponding time lags that are stored within the class to facilitate further analysis.

### 2.8. Report Generation

**Figure 3:**
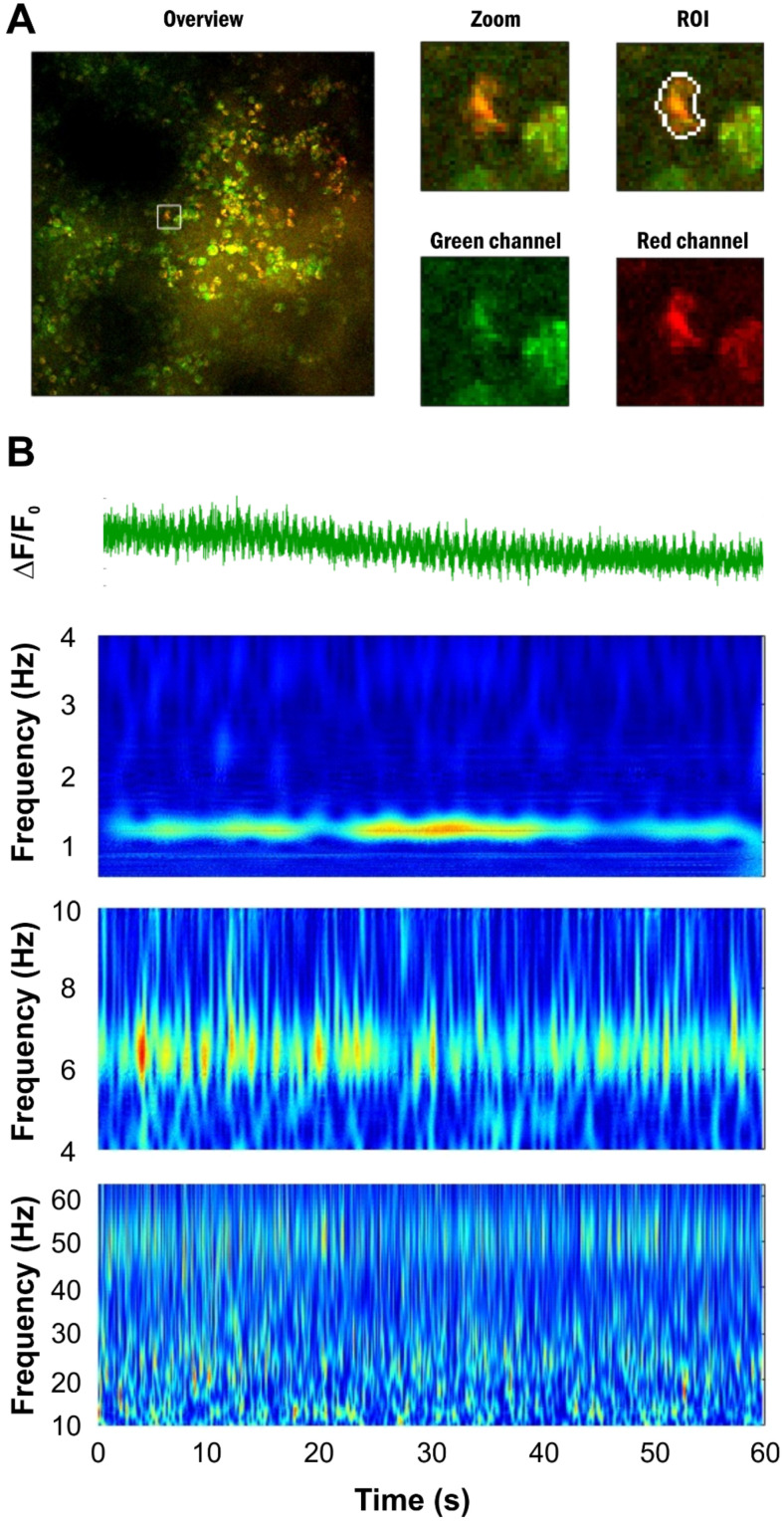
Automatic report created for individual cells. (**A**) Location of a given cell is marked by a white rectangle on the whole field of view (left). A higher magnification image of the cell together with ROI boundary and separate images of the green and red channel fluorescence intensity are also provided (right). (**B**) ΔF/F_0_ trace of the given ROI (top) and wavelet analysis of this trace in different frequency bands. The automatically generated report contains all the information for all the identified ROIs.

**Figure 4:**
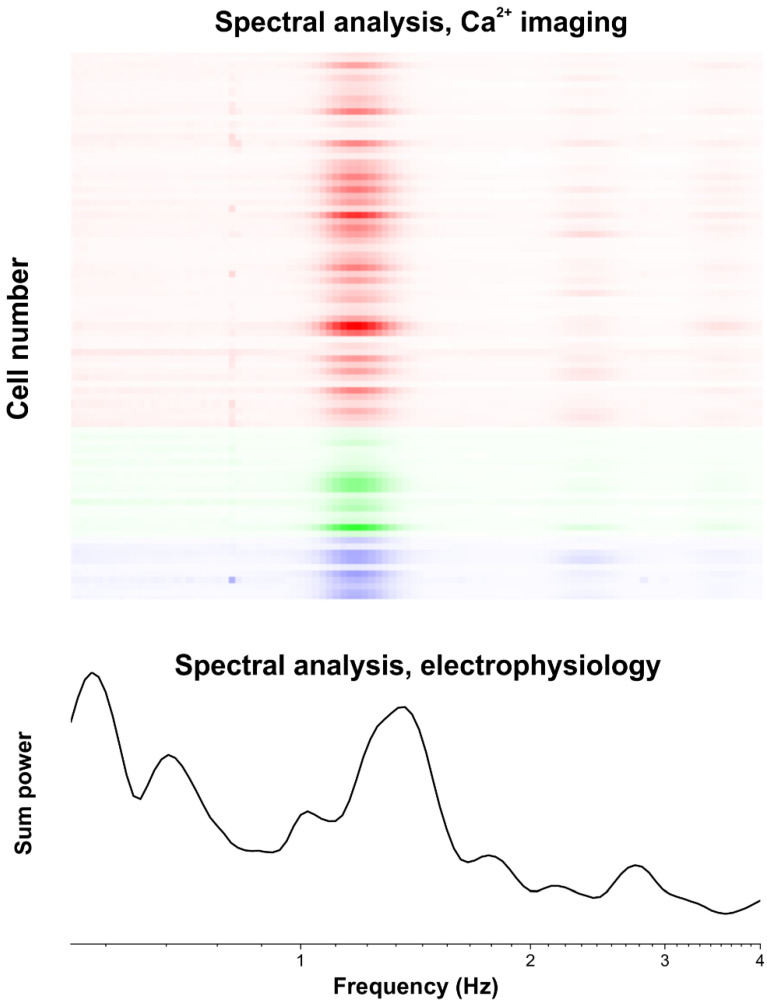
Automatic report created for network activity. (**top**) Spectral analysis of all cells within an imaging session. Each line shows the wavelet coefficients summed at different frequencies for a given cell. Astrocytes are shown in red, neurons in green and regions not identified as cells are shown in blue. Scale of the x-axis is the same as in (**bottom**). (**bottom**) Power distribution of the simultaneously measured field potential signal shows high correlation with the imaging frequency components. The report, automatically generated by the ImagingDataSet class, contains this information for all frequency bands for a large number of corresponding imaging sessions.

When analyzing a large dataset, it is crucial to have the ability to summarize the results in an easily comprehensible manner. The ‘reportCellData’ function generates a comprehensive report of the main results, formatted as a Word document. The report contains detailed information on imaging settings, cell types, and electrophysiological recordings, supplemented with graphical representations of the wavelet transforms and ΔF/F_0_ traces. In addition, it provides segmented imaging data, distinguishes between astrocytes, neurons, and non-cellular regions, with images and analyses for individual cells. Examples of automatically generated reports are shown in Figure 3 and Figure 4 for an in vivo Ca^2+^ imaging measurement of visual cortex neurons and astrocytes under ketamine/xylazine anesthesia, which is known to induce permanent slow wave activity [16,17], and it is demonstrated by simultaneously obtained Ca^2+^ imaging and electrophysiological data (Figure 4).

### 2.9. Working with Multiple Datasets

To facilitate the analysis of multiple measurements, the ImagingDataSet class provides the ability for handling a large number of results calculated using the ImagingData class. The ‘loadDataFromFolder’ function imports .MAT files containing previously analyzed results. Subsequently, the ImagingDataSet class provides various ways for applying the same function to each result set for monitoring activity changes on a longer timescale or to compare the calcium activity under different conditions.

## 3. Discussion

The FluoAnalysis MATLAB toolbox offers a sophisticated and user-friendly solution for the comprehensive analysis of calcium imaging data integrated with electrophysiological recordings. By organizing its functionalities into well-structured MATLAB classes with accessible GUIs, the toolbox facilitates a streamlined and efficient workflow. This workflow encompasses essential steps such as data loading, ROI identification, cell classification, fluorescence intensity calculation, spectral analysis, and report generation, all of which can be executed manually through the GUI or automated for a high-throughput analysis.

Researchers can use the toolbox for investigating the dynamics of neuronal and astrocytic activity on both cellular and network levels to explore the cellular mechanisms underlying physiological and pathophysiological functions. It can also be used to assess the effects of drugs on neuronal and astrocytic activity, understand drug mechanisms, and identify potential therapeutic targets. The analysis of even large imaging datasets may enable the high-throughput screening of compounds.

Current calcium imaging analysis tools all offer basic functionalities, like cell segmentation and spike detection, which make them suitable to extract the essential features of neuronal calcium signaling. However, state-of-art software, like CaImAn [3], EZcalcium [2], and Suite2p [4] usually put special focus on a feature that make them extremely useful in achieving specific goals (Table 1). In terms of automation, CaImAn and Suite2p both offer fully automated workflows, making them ideal for handling large-scale datasets with minimal user intervention. However, a certain level of programming experience in either MATLAB or Python is required to use them efficiently, posing a barrier for those less familiar with coding. In contrast, Ezcalcium and FluoAnalysis provide a user-friendly GUI, making it more accessible to researchers without extensive programming skills. CaImAn is particularly strong in providing advanced algorithms for non-rigid motion correction and automated cell segmentation, while Suite2p excels in speed, leveraging GPU acceleration to quickly process data from thousands of neurons. FluoAnalysis is distinguished in three areas, which makes it unique in relation to other calcium imaging analysis software programs. It can integrate electrophysiological data and process these data simultaneously with imaging results. Furthermore, FluoAnalysis focuses on the differentiation between neurons and astrocytes, enabling the parallel analysis of calcium signaling of both cell types. Most importantly, FluoAnalysis pays attention to analyzing oscillatory signals in the frequency domain rather than detecting individual peaks. These features make FluoAnalysis particularly useful in investigating network activity.

## 4. Conclusions

In conclusion, the FluoAnalysis MATLAB toolbox provides a powerful and versatile platform for the comprehensive analysis of calcium imaging and electrophysiological data, supporting a wide range of research applications in neuroscience. The toolbox is especially focused on the analysis of oscillatory activity, occurring at the network level, simultaneously measured in large number of cells. The analysis pipeline offered by the FluoAnalysis toolbox can be fully automated but can also be used by researchers with no previous programming skills, through a user-friendly graphical user interface.

## 5. Materials and Methods

### 5.1. Software Setup

The FluoAnalysis toolbox is freely available to download from GitHub and the Matlab File Exchange website, along with sample data that demonstrate the capability of the software. It can be installed by simply adding the downloaded files to the MATLAB path. After typing ‘FluoAnalysis’, a GUI will load which can run all the individual modules described here. The different functions can also be applied by the <instance name>.<function name> command. Programming skills are not required to use the FluoAnalysis toolbox. However, a basic understanding of the Matlab programming language may help with fine-tuning various parameters, hardcoded in the scripts (e.g., to change the wavelet type used in the wavelet analysis).

FluoAnalysis was tested on MATLAB versions from R2020a to R2024a. The following MATLAB toolboxes are required to be installed to use FluoAnalysis: Control System, Signal Processing, Image Processing, Statistics and Machine Learning, Wavelet, Database, Bioinformatics, and Computer Vision.

### 5.2. In Vitro Ca^2+^ Imaging

Ca^2+^ imaging was performed using a Femto2D two-photon microscope (Femtonics, Budapest, Hungary) equipped with a 10× water immersion objective (N.A. 0.30). Acute hippocampal/cortical slices were incubated right after slicing by changing the normal ACSF solution in the interface type chamber to an ACSF containing 1 µM of the astrocyte-specific marker SR101 for 20 min at 37 °C [18,19]. Brain slices were either prepared from rats stably expressing the Ca^2+^-sensitive fluorescent protein GCaMP2, or were incubated with 10 µM of the calcium-sensitive fluorescent dye OGB-1 AM in ACSF at 37 °C for 1 h in the dark under a continuously oxygenated atmosphere for another one hour after the initial one hour of incubation in the interface-type chamber [20]. Slices were transferred into a submerge-type recording chamber mounted on the stage of the microscope and were superfused with oxygenated ACSF (3 mL/min, ~30 °C). Images were taken at the traditional sampling frequency of 1 Hz (Figure 1) or at a much higher rate of 68 Hz (Figure 2) for identifying high-frequency components in neuronal and astrocytic Ca^2+^ signals. All fluorescent compounds were excited at 900 nm by a MaiTai laser source (Spectra-Physics, Milpitas, CA, USA) or at 920 nm by a FemtoFiber ultra 920 laser source (Toptica Photonics AG, Graefelfing, Germany). Emitted fluorescence was monitored at 475–575 nm (OGB-1) and 600–700 nm (SR101).

### 5.3. In Vivo Ca^2+^ Imaging

Wistar rats (>300 g), prepared with a cranial window, were injected with 160 µM OGB-1 and 140 µM SR-101, suspended in 10 µL ACSF, through an implanted epidural cannula, 150 min before the imaging sessions. All measurements were performed under ketamine–xylazine anesthesia (Figure 3 and Figure 4). Head fixation was achieved using an implanted aluminum plate or a stereotaxic frame. With the head fixed, the rest of the body was rotated so the animal was laying on its left side. This helped to minimize any movement artifacts resulting from breathing during imaging. Imaging data were recorded from a 100–300 µm depth, corresponding to layer 2 of the V1 primary visual cortex, using a Femto2D two-photon microscope (Femtonics, Budapest, Hungary), equipped with a 10× water immersion objective. Cells were excited by a 920 nm laser (FemtoFiber ultra 920, Toptica Photonics AG, Graefelfing, Germany). OGB-1 fluorescence was detected at 475–575 nm, and SR-101 at 600–700 nm. A total of 85 cells were imaged simultaneously in 60 s long line-scan sessions at a sampling frequency of 125 Hz. Changes in OGB-1 fluorescence were detected in the line-scanning mode to achieve a high sampling frequency. The scanning path was determined automatically based on the position of identified cells. Importantly, two crossing line paths were applied to each cell to minimize the divergence of the scan head movements from the set path due to its inertia. The scanning order of the identified cells was optimized using the travelling salesman method.

## Figures and Tables

**Table 1:** Comparison of features of the FluoAnalysis toolbox with other calcium imaging analysis tools.

Feature	CaImAn	Ezcalcium	Suite2p	FluoAnalysis
Graphical User interface	−	+	−	+
Automation Level	Fully automated	Semi-automated	Fully automated	Fully automated
Motion Correction	+	+	+	Limited; basic motion correction capabilities
Cell Segmentation	+(sophisticated, automatic)	+(automatic and manual)	+(sophisticated, automatic)	+(automatic and manual)
Handling multiple cell types	−	−	−	Differentiate between neurons and astrocytes
Integration with electrophysiological data	−	−	−	+
Spike detection	+	+	+	+
Spectral analysis	−	−	−	+

## Data Availability

The FluoAnalysis toolbox is freely available to download from GitHub (https://github.com/hejalaszlo/FluoAnalysis, accessed at 16 August 2024) and the Matlab File Exchange website (https://www.mathworks.com/matlabcentral/fileexchange/169801-fluoanalysis, accessed at 16 August 2024) along with sample data that demonstrate the capability of the software.
